# Chromatin remodeling in lymphocytic function and fate: the multifaceted roles of SWI/SNF complex

**DOI:** 10.3389/fimmu.2025.1575857

**Published:** 2025-04-24

**Authors:** Renjie Miao, Yun Liu, Shuo Shen, Wenxin Wang, Shengjun Wang

**Affiliations:** ^1^ Affiliated Third Hospital of Zhenjiang to Jiangsu University, Zhenjiang, Jiangsu, China; ^2^ Affiliated Hospital of Jiangsu University, Zhenjiang, Jiangsu, China; ^3^ School of Medicine, Jiangsu University, Zhenjiang, Jiangsu, China

**Keywords:** SWI/SNF complex subunits, chromatin remodeling function, lymphocytic biology, clinical treatments and applications, chimeric antigen receptor, immunotherapy

## Abstract

The Switch/Sucrose Non-Fermentable (SWI/SNF) chromatin remodeling complex comprises 10-15 subunits, which modulate the arrangement, location, or conformation of nucleosomes to upregulate chromatin accessibility. During lymphocytic differentiation and functional development, the SWI/SNF complex exerts its effects by binding to specific transcription factors (TFs) or DNA sequences via its subunits, which are thereafter recruited to the promoter or enhancer regions of target genes, rendering each subunit crucial wherein. The loss of individual subunits during lymphocytic differentiation not only disrupts the targeting of the SWI/SNF complex but also impairs its chromatin remodeling function, ultimately resulting in altered differentiation of immature lymphocytes, dysfunction of mature lymphocytes, and injured immune responses. Therefore, in this paper, we focus on TFs interacting with SWI/SNF complex subunits in lymphocytes, and summarize the effects of the loss of specific subunits of the SWI/SNF complex on lymphocytic differentiation and function, as well as the modification in the expression of key genes. We also summarize the potential clinical treatments and applications targeting the loss of SWI/SNF complex subunits, and focus on the application in Chimeric Antigen Receptor (CAR) technology. In conclusion, the SWI/SNF complex is a key regulatory factor in lymphocytic biology, involved in fundamental cellular processes and closely associated with hematological diseases and immune dysfunction. However, the specific roles of SWI/SNF complex subunits in different lymphocytic subpopulations remain unclear. Future clarification of the specific functions of these subunits in different lymphocytic subsets is expected to promote the development of immunotherapy and personalized therapy.

## 1 Introduction

### 1.1 Overview of SWI/SNF complex

The Switch/Sucrose Non-Fermentable (SWI/SNF) chromatin remodeling complex is a highly conserved, ATP-dependent complex, with ample presence in eukaryotes. Its core function is to upregulate chromatin accessibility via nucleosome sliding, displacement, and removal, thus affecting gene expression and chromosomal stability (1–4). Therefore, the SWI/SNF complex plays a role in the differentiation of majority of cells (5). The SWI/SNF complex consists of approximately 10-15 subunits, and its overall function is highly coordinated with the specific temporal and spatial roles of individual subunits. Certain subunits are expressed exclusively at specific stages of lymphocytic differentiation. In most cases, the SWI/SNF complex collaborates with specific transcription factors (TFs) via its subunits to stabilize their binding to DNA and recruit other cofactors to enhance their activity. Additionally, the complex is targeted to regions such as promoters, enhancers, and histone acetylation sites, contributing to the transition of chromatin in the target gene from a compact to an open state, which promotes the further binding of multiple TFs and RNA polymerase II to chromatin, leading to the upregulated gene expression, cell differentiation, proliferation, function, and cell cycle. In some cases, the SWI/SNF complex can directly bind to specific DNA sequences or histone acetylation sites via its subunits. Therefore, the loss of any subunit disrupts the targeting or overall stability of the SWI/SNF complex. These subunits form three main subtypes by diversified combinations (6–10): canocical BAF (cBAF), polybromo BAF (PBAF), and non-canocical BAF (ncBAF), each with specific structural and functional roles to meet various biological functions (**Table 1**). In conclusion, the SWI/SNF complex plays a central role in regulating cellular functions by chromatin remodeling. Its diversity and complexity make it a crucial target for research on cell development, immune regulation, and disease treatment.

**Table 1:** SWI/SNF complex subunits.

Subunit Type	Subunit Name
Common Subunit	SMARCA4 (BRG1, SWI/SNF related, matrix associated, actin dependent regulator of chromatin, subfamily a, member 4)/ SMARCA2 (BRM)
	SMARCB1 (BAF47/INI1/SNF5)
	SMARCC1 (BAF155/SRG3)
	SMARCC2 (BAF170)
	SMARCE1 (BAF57)
	DPF1/2/3 (BAF45B/D/C, Double PHD fingers 1/2/3)
	SMARCD1/2/3 (BAF60A/B/C)
	ACTL6A/B (BAF53A/B, Actin-like 6A/B/C)
	ACTB (β-actin)
	BCL-7A (B-cell lymphoma 7A)
cBAF Specific	ARID1A (BAF250a, AT-rich interactive domain 1A)
	ARID1B (BAF250B)
PBAF Specific	PBRM1 (Polybromo 1)
	PHF10 (PHD finger protein 10)
	BRD7 (Bromodomain Containing 7)
	ARID2 (BAF200)
ncBAF Specific	BRD9
	GLTSCR1/GLTSCR1L (BAF45C/A, BRD4 interacting chromatin remodeling complex associated protein 1/1 like)

### 1.2 Basic process of lymphocytic development

Lymphocytes, including T cells, B cells, and NK cells, originate from hematopoietic stem cells (HSCs) and undergo multiple developmental stages (11). Different differentiation stages include various key genes (12–16). Throughout these stages, the SWI/SNF complex upregulates the transcriptional levels of key factors involved in lymphocytic differentiation and function by binding to specific DNA sequences, histone acetylation sites, or interacting with specific transcription factors (TFs), thereby affecting the expression of a series of genes.

### 1.3 Current research on SWI/SNF complex in lymphocytes

The SWI/SNF complex plays a crucial role in maintaining normal immune cell development and responses (7, 17–19). However, the specific roles of its subunits in lymphocytes have not been thoroughly explored. Recent advancements in CRISPR gene editing and epigenomics have provided higher resolution for understanding the specific regulatory roles of its subunits. Prior studies focused primarily on the immature stages of lymphocytes, such as HSCs and common lymphoid progenitors (CLPs). Recent researches have shifted toward the roles of SWI/SNF complex subunits in terminal differentiation and functional regulation of mature lymphocytes, gradually applying these regulatory mechanisms to disease treatment (7, 20–22).

Most reviews available on the SWI/SNF complex focus on various cancers (2, 23). Some reviews focus on hematologic diseases such as leukemia and lymphoma, while the latest reviews emphasize the overall role of the SWI/SNF complex in lymphocytic development and inflammatory diseases (2, 23, 24). This review specifically addresses changes in key differentiation genes, effector genes, exhaustion genes, cell cycle genes, and memory phenotypes following the loss of SWI/SNF complex subunits in lymphocytes, and provides a summary of their potential treatments and applications (**Table 2**). At the same time, the existing research tools and research models are summarized (**Supplementary Table**).

**Table 2:** Effect of subunit loss on lymphocytic differentiation and function.

Subunit Loss	Cell	Factors of collaboration or co-localization	Up-regulated genes	Down-regulated genes	Impact on cell function	Ref
ARID1A	HSC and CLP	GATA2, RUNX1, PU.1	*/*	*GATA2, CEBPA, CD34, IL6R, CSF1*	Impairing early lymphocytic differentiation	(30)
	T cell (DN1-DN4)	GATA3, TCF-1, RUNX1, BCL-11B	*/*	*BCL-11B, TCF7, IL2RA, RAG2, CD8A, CD44*	Impairing early lymphocytic differentiation	(20)
	CD4 + T cell	MATR3	*/*	*TOX*	Increasing cell persistence	(33)
	CD8 + T cell	AP-1	*IFNG, TNF*	*PDCD1, LAG3, CCL5*	Increasing cell persistence	(22)
	CD8 + T cell	SMARCD2, SMARCA4, C-MYC	*CXCR3, CD27, CD62*	*GZMB, IL2RA, TBX21*	Reducing cell exhaustion and increasing memory phenotype	(21)
	CD8 + T cell	ETS1, T-BET, BATF, IRF4	*TCF7, CCR7, ID3, MYB*	*TBX21, ZEB2, GZMB, BATF, BHLHE40*	Reducing cell exhaustion and increasing memory phenotype	(7)
	Mature B Cell	PU.1, NF-ΚB, SPIB, IRF4/8, STAT3, RUNX1, PAX5, CTCF	*IL1Β, IL6, IFNG, CCL2, CCL3, CCL8, CXCL10, IFITM3, IFIT1, MX1*	*CD72, TIGIT, SPRY2, PRDM1, IRF4, BCL-6*	Impairing plasma cell differentiation	(34)
	Mature B Cell	PU.1, NF-κB, SPIB, IRF4/8, STAT3, PAX5, CTCF	*KLF2, CD38*	*CD40, STAT3, IL-2, IL-4, IL-6*	Impairing plasma cell differentiation	(35)
ARID1B	HSC and CLP	RUNX, SPIB, CTCFL	*/*	*CSF1R, CSF1, CD34, FLT3, IL1R1*	Impairing early lymphocytic differentiation	(40, 41)
ARID2	HSC and CLP	NF-ΚB, PU.1, SPIB, SPIC	*TLR4, IL1, PU.1, MPP3*		Impairing early lymphocytic differentiation	(45)
	CD8^+^T cell	IRF5/6, BLIMP-1, TCF-1	*GZMB, CD127, CDC25A, CCND1, AURKA, KI67, CDK1, E2F1*	*BIK, FAS, IRF5, IRF6*	Reducing cell exhaustion and enhancing immune effects	(54, 56)
SMARCB1	Treg	SMARCA4, ARID1A	*CASTOR1*	*/*	Impairing cell function and metabolism	(59)
	CD8^+^ T cell	Rb	*/*	*/*	Impairing early lymphocytic differentiation	(57)
SMARCC1	HSC and CLP	/	*/*	*FOS, JUN, IL2, IL4, IFNG, IL17*	Impairing early lymphocytic differentiation	(71, 72)
	HSC and CLP	CEBPα, GATA1, E2A	*/*	*CCR2, ESAM, GATA2*	Impairing early lymphocytic differentiation	(73)
	TH17	RORγt	*/*	*IL17A, IL17F, IL23R*	Impairing cell differentiation and function	(77)
	GC Tfh	BCL-6	*PRDM1*		Impairing cell differentiation and destroying GC	(81)
SMARCD1	HSC and CLP	E2A	*/*	*RAG1, RAG2, IL7R*	Impairing lymphocytic differentiation	(82)
	Treg	RUNX1	*/*	*CCR9*	Damaging cell migration ability	(83)
SMARCD2	CD8 + T cell	C-MYC, ARID1A, SMARCA4	*CXCR3, CD27, CD62L*	*GZMB, IL2RA, TBX21*	Reducing cell exhaustion and increasing memory phenotype	(21)
SMARCA4	HSC and CLP	SMARCD2, DPF2	*MAC1, ITGAM*	*KIT, MYC*	Impairing early lymphocytic differentiation	(85, 91–93)
	HSC and CLP	/	*/*	*KLF2A*	Destroying the NO microenvironment	(90)
	Pro-T Cell, Pro-B Cell, CD4^+^ T Cell	/	*/*	*GATA3, TBX21, STAT5A, ETS1, FLI1*	Impairing early lymphocytic differentiation	(94)
	Pre-B Cell	E2A, EBF1, IKZF1, PAX5	*/*	*MYC*	Impairing early lymphocytic differentiation	(72, 95)
	CD4 + T cell	EZH2	*/*	*CD274*	Reducing cell exhaustion	(98)
	CD8 + T cell	AP-1, NFAT, NF-ΚB	*/*	*IFNG, TNF, IL2*	Impairing cell function	(97)
	CD8 + T cell	BATF, IRF4, MYB	*/*	*TOX, NR4A, ENTPD1, CXCL13, ITGA2, HAVCR2, TIGIT, HNF1B*	Increasing cell persistence	(97)
	TH1	/	*/*	*IL12RB2*	Impairing cell function	(99, 100)
	Mature B Cell	PU.1, IRF, NF-ΚB	*BCL6*	*/*	Impairing the differentiation of plasma cell and Memory B cell	(102, 103)
	Immature B Cell	/	*PPP2R1A*	*MYC, BCL-2*	Impairing early lymphocytic differentiation	(109)
SMARCA2	CD4 + T cell	EZH2	*CD274*	*/*	Accelerating cell exhaustion, promoting tumor immune escape	(98)
	TH1	WASP	*/*	*IL2, IL4, IFNG, IL17*	Impairing cell function	(101)
PBRM1	HSC and CLP	/	*CDKN1A*	*/*	Impairing early lymphocytic differentiation	(115)
	Th2	/	*IL10*	*/*	Promote the expression of IL-12	(116)
BRD7	CD8 + T cell	/	*/*	*TBX21*	Impairing function	(124)
BRD9	Immature B Cell	CTCF	*/*	*/*	Impairing early lymphocytic differentiation	(125)
	Treg	FOXP3	*/*	*CTLA4, ICOS, TIGIT*	Impairing cell function	(19)
ACTL6A	HSC and CLP	/	*/*	*MYC, RUNX1*	Impairing early lymphocytic differentiation	(130)
PHF10	HSC and CLP	/	*/*	*GATA2, CEBPA, PU.1*	Impairing early lymphocytic differentiation	(134)

## 2 Specific regulatory roles of SWI/SNF subunits in lymphocytes

### 2.1 ARID protein family

ARID1A, ARID1B, and ARID2 are key epigenetic regulators and core subunits of the SWI/SNF complex. They play roles in various lymphocytic functions by means of collaboration with multiple TFs. Their loss during the development of immature lymphocytes can lead to abnormal lymphocytic development or the occurrence of various hematologic diseases. However, in mature T cells, intervention in ARID1A and ARID2 can effectively improve the T cell exhaustion (Tex) state to improve immunotherapy. Therefore, in-depth research on these factors in different diseases will help develop novel immunotherapy strategies targeting them or their cooperating TFs.

#### 2.1.1 ARID1A

ARID1A is a core subunit of cBAF, closely related to immune responses in the body, and most of the targeting localization of cBAF relies on ARID1A. On one hand, the deletion of ARID1A in lymphocytes can directly impair early differentiation and alter terminal fate decisions (7). On the other hand, in tumors, the loss of ARID1A can lead to low differentiation of cells, upregulated PD-L1 expression, suppression of CD8^+^ T cell activity, and enhanced tumor malignancy (25, 26). Interestingly, studies have identified that ARID1A deletion has a dual role in promoting cancer and enhancing immune responses. Its deletion also leads to the escape of DNA breakage in tumor cells, producing more single-stranded DNA (ssDNA), which is then recognized by the cyclic GMP-AMP synthase (cGAS) protein, activating the cGAS-STING signaling pathway to induce IFN-I production (27, 28). This attracts immune cells, especially CD8^+^ T cells, enhancing anti-tumor immune responses. In an HIV-1 infection model, the deletion of ARID1A can also induce the activation of latent HIV-1, enhancing the recognition and killing functions of CD8^+^ T cells and NK cells (29).

During the immature lymphocyte stage, the loss of ARID1A in early HSCs impairs its interaction with TFs such as GATA2, RUNX1, and PU.1, disrupting the targeting localization and function of the SWI/SNF complex. This directly leads to the impairment of transcriptional level of key hematopoietic genes like GATA2, CEBPA, IL6R, and CSF1, damaging the frequency and function of HSCs. Ultimately, this tiptipleads to impaired lymphocytic differentiation and decreased population of peripheral mature lymphocytes (30). Meanwhile, studies show that in the DN1-DN4 (Double negative stage) of T cell differentiation, ARID1A co-localizes with GATA3, TCF-1, RUNX1, and BCL-11B in chromatin regulatory regions, targeting the recruitment of the SWI/SNF complex while enhancing the binding and activity of these key TFs. The loss of ARID1A directly leads to reduced expression of key T cell differentiation and development genes such as BCL11B, TCF7, and CD44, thereby regulating early T cell development (20). Thus, the loss of ARID1A impacts the early differentiation and maturation of T cells.

In mature T lymphocytes, ARID1A is closely involved in the transition between the effector, memory, and exhaustion states of CD4^+^ and CD8^+^ T cells. After effector T cells perform their function, they enter either an exhaustion state or a memory state. TOX is a specific regulator for exhausted T cells that controls a range of genes of exhaustion phenotype and functional maintenance (31, 32). Recent research in CD4^+^ T cells has shown that MATR3, as a key regulator of CD4^+^ T cell function in a liver cancer model, recruits the ARID1A-containing cBAF complex to enhance chromatin accessibility at the TOX promoter region, thereby exacerbating CD4^+^ Tex (33). In recent studies of CD8^+^ T cells in chronic tumor models, it was also reported that ARID1A cooperates with AP-1 to target the recruitment of cBAF to exhaustion gene chromatin regions, enhancing the expression of exhaustion genes such as PDCD1 and LAG3. Once ARID1A is lost, the expression of exhaustion genes is suppressed, making TF AP-1 more inclined to activate effector genes such as IFN-γ and TNF-α (22). However, two recent studies in acute infection models reported some differences compared to tumor models, particularly in the regulation of effector T cells (7, 21). One study described that ARID1A can also primarily bind with SMARCA4 and C-MYC at the promoters and enhancers of T cell effector genes such as GZMB and TBX21. When ARID1A and MYC are co-suppressed, effector T cell generation is reduced, whereas the generation of memory T cell genes is promoted (21). The other study further demonstrated that the loss of ARID1A enhances the differentiation of memory precursor cells, leading to decreased chromatin accessibility at effector genes, while increasing chromatin accessibility at memory genes (7).

During the determination of mature B cell fate, cBAF, via ARID1A, collaborates with TFs of differentiation and immune function, such as PU.1 and NF-κB, to enhance the expression of genes in B cell differentiation, while inhibiting the expression of excessive inflammatory factors, chemokines, and interferon response genes. This helps maintain the stability of the GC and efficient immune responses (34). Therefore, the loss of ARID1A directly impairs the differentiation pathway of GC (Germinal center) B cells, while also triggering a potent inflammatory response, attracting neutrophils and inflammatory monocytes to infiltrate and disrupt the differentiation of GC B cells, which results in increased generation of immature memory B cells and decreased generation of mature plasma cells. In particular, in the presence of oncogenes such as BCL2, B cells are more likely to transform into more aggressive follicular lymphoma or diffuse large B cell lymphoma (34, 35).

In summary, ARID1A plays a crucial role in T cell development from early stages to the formation of function and memory. These studies support targeting ARID1A for the treatment of cancer, immune diseases, and viral infections. The loss of ARID1A in the immature stage of lymphocyte directly leads to the differentiation disorder. The regulatory effect of ARID1A loss in inhibiting Tex and promoting memory T cell generation has been recognized by several studies. However, the dual role ARID1A plays in effector T cell function after its loss depends on differences in experimental models and time dimensions. Current research suggests that in chronic tumor models, ARID1A loss enhances effector T cell function and improves the persistence of anti-tumor immunity. However, in acute infection models, the loss of ARID1A damages the function of effector T cells. In B cells, precise intervention of ARID1A is crucial for maintaining normal B cell function and preventing lymphoma development. Therefore, a deeper understanding of ARID1A in different cell types and diseases can enable more accurate treatment regimens targeting specific disease states and provide novel perspectives and potential intervention sites for future research and clinical applications.

#### 2.1.2 ARID1B

There may be functional overlap between ARID1A and ARID1B, both being synthetic lethal targets (30, 36, 37). ARID1B plays a crucial role in compensating for ARID1A loss. While ARID1A mutations are well-established in tumors, ARID1B mutations are also frequently reported in cancers, albeit with a lower frequency (38, 39). Compared to ARID1A, the modified chromatin accessibility due to ARID1B loss are less pronounced. While ARID1B is not essential for homeostatic hematopoiesis, it is equally important for the regenerative capacity of HSCs. In the case of ARID1B loss, chromatin accessibility at binding sites for RUNX, SPIB, and other factors is suppressed, suggesting that ARID1B-mediated cBAF may be recruited to these regions. This leads to the suppression of hematopoietic gene expression upon ARID1B loss, weakening the regenerative capacity of marrow and indirectly impairing the formation of subsequent immature lymphocytes (40, 41).

Thus, it is clear that ARID1B primarily functions in HSCs, with the loss of ARID1B leading to early differentiation defects in lymphocytes. In cases of hematopoietic system damage (such as after bone marrow transplantation), monitoring ARID1B can help prevent acute bone marrow failure.

#### 2.1.3 ARID2

ARID2 is a key member of PBAF, replacing ARID1A/B in cBAF, with some functional overlap (8, 42). The ARID2-mediated PBAF plays an important role in HSCs and prevents leukemia (43, 44). Similar to ARID1A, ARID2 can also affect the development of immature lymphocytes by cooperating with PU.1 and NF-κB. The loss of ARID2 leads to the activation of inflammatory pathways and impaired lymphocytic differentiation, while enhancing myeloid differentiation (34, 35, 45). Recent studies have reported that ARID2 plays a crucial role in balancing the effector and exhaustion states of mature T cells in chronic viral infections and cancer. The PBAF complex may maintain immune balance by limiting T cell proliferation and effector functions.

Tex comprises three functionally distinct subpopulations: Tex Progenitor (Tex Prog), Tex Intermediate (Tex Int), and Tex Terminal (Tex Term) (46–48). TCF7, PRDM1, TBX21, and EOMES are closely associated with the conversion between Tex subpopulations (49–53). ARID2, via the PBAF complex, mediates modification in the expression levels of TCF7, TOX, and PRDM1 to control the transition between different Tex subpopulations (54). The loss of ARID2 also enhances the response to PD-1 pathway blockade, while decreasing the expression of apoptosis and IFN signaling pathway genes. In contrast, genes of proliferation and effector functions are upregulated, and cell cycle genes such as CCND1 and KI67 are also upregulated. This promotes the differentiation of CD8^+^ T cells from exhausted precursors to effector T cells, reduces the formation of exhausted T cells, and enhances antiviral and antitumor immune responses (54–56).

Therefore, targeting ARID2 can enhance the proliferation capacity and antitumor activity of CD8^+^ T cells in chronic infections and cancer, thereby improving the effectiveness of immunotherapy. Additionally, the loss of ARID2 leads to reduced differentiation of early lymphocytes and increased the likelihood of leukemia development.

### 2.2 SMARC protein family

#### 2.2.1 SMARCB1

SMARCB1 is widely present in all SWI/SNF complexes, where it maintains the stability of the complex. Early studies have revealed that the loss of SMARCB1 can directly lead to the formation of T cell lymphoma (57). mTORC1 is a key signaling pathway that promotes cell growth, metabolism, and survival, and has a significant impact on T cell function and differentiation (58). Recent studies have also revealed that Treg cells with SMARCB1 loss exhibit upregulation of CASTOR1, which leads to impaired mTORC1 activation, interfering with the function and metabolism of Treg cells. This may be attributed to the disruption of the cooperation between SMARCB1, SMARCA4, and ARID1A owing to SMARCB1 loss, leading to overall dysfunction of the SWI/SNF complex (59).

Research on SMARCB1 in lymphocytes is currently limited. It is known that the loss of SMARCB1 damages the overall function of the SWI/SNF complex and interferes with Treg cell function and metabolism (59). However, recent studies in tumors have documented that the loss of SMARCB1 leads to abnormal assembly of the SWI/SNF complex and abnormal expression of EZH2. Targeting and inhibiting EZH2 and DCAF5 shows therapeutic potential (60, 61). EZH2 also plays a role in the development and function of T cells and B cells (62, 63). Therefore, clarification of the mechanism of interaction between SMARCB1 and EZH2 in lymphocytes in the future is expected to provide novel treatment options for patients with lymphocyte-related diseases.

#### 2.2.2 SMARCC1/2

SMARCC1, as a core scaffold protein of the SWI/SNF complex, helps maintain the stability of the complex and often collaborates with other factors to promote inflammation gene expression and lymphocytic differentiation. Therefore, the loss of SMARCC1 significantly damages the stability of the SWI/SNF complex and interferes with the production of inflammatory factors in the body (64, 65). Recent studies have revealed that SMARCC1 and SMARCC2, unlike other subunits, play dual roles in cancer, both promoting and suppressing tumor growth (66–70).

During the immature lymphocytic stage, prior studies reported that the loss of SMARCC1 leads to impaired stability of the SWI/SNF complex, which directly causes impaired expression of genes such as FOS and IL2, resulting in lymphocytic developmental defects (71, 72). Recent studies have further revealed that after the loss of SMARCC1, the chromatin accessibility of TFs such as CEBPα, GATA1, and E2A significantly decreases, leading to downregulation of HSC differentiation genes and a dramatic reduction in B cells and CD8^+^ T cells (73). The loss of SMARCC2 also impairs the differentiation of embryonic pluripotent stem cells (74).

These findings demonstrate the “scaffold” function of SMARCC1/2 in the SWI/SNF complex. SMARCC1 can also collaborate with specific TFs to target the recruitment of the SWI/SNF complex to specific chromatin regions. Firstly, in the formation of TH17 cells, recent studies have revealed that the SWI/SNF complex coordinates TH17 cell differentiation. TH17 cells are a subset of mature T cells that can secrete IL17 and its overactivation can enhance inflammatory responses and autoimmune damage (75, 76). The differentiation of TH17 cells depends on the master TF RORγt, which recruits the SWI/SNF complex by binding to SMARCC1 to modify histones and enhance chromatin accessibility at the TH17 signature gene regions. In the absence of SMARCC1, there is specific downregulation of RORγt target genes such as IL17A, leading to impaired TH17 cell differentiation (77, 78).

Additionally, the SWI/SNF complex can also promote the formation of GC. GCs are the foundation for plasma cell formation, where Tfh cells and GC B cells interact to maintain the stability of GCs (79, 80). Studies have found that SMARCC1 can directly interact with BCL-6, targeting the recruitment of the SWI/SNF complex to the BCL-6 response element region of the PRDM1 gene, inhibiting PRDM1 expression to maintain GC. SMARCC1 deficiency can lead to GC disruption and reduced Tfh cell expression (81).

In summary, SMARCC1 and SMARCC2 serve as both scaffold and functional subunits in the SWI/SNF complex. They have a significant impact on lymphocytic activation, proliferation, and differentiation (particularly TH17 cell differentiation). SMARCC1 also plays a core role in maintaining GC formation in B cell-mediated humoral immunity. However, research on SMARCC1 and SMARCC2 in lymphocytes is currently limited, and their specific mechanisms and roles in various lymphocytic subpopulations require further investigation.

#### 2.2.3 SMARCD1/2

SMARCD1 and SMARCD2 are shared subunits of the three SWI/SNF complexes and are also core regulators in lymphocytic lineage differentiation.

SMARCD1 collaborates with TFs such as EA2 to promote the expression of key lymphocytic lineage genes (82). The loss of SMARCD1 leads to a reduction in immature T cells in the bone marrow and thymus, with a significant decrease in the number of peripheral mature lymphocytes (82). SMARCD1 can also co-localize with RUNX1 at the enhancer region of the CCR9 to increase CCR9 expression, improving the migration ability of Treg cells to inflammatory sites and thereby indirectly enhancing immune responses (83).

Compared to SMARCD1, SMARCD2 is more focused on regulating myeloid cell differentiation (84). Studies in mature lymphocytes show that the loss of SMARCD2 can impair early lymphocytic lineage differentiation and significantly promote the generation of memory T cells (21, 85).

Overall, SMARCD1 and SMARCD2 play pivotal roles in the differentiation of lymphocytes and myeloid cells. SMARCD1 promotes lymphocytic differentiation by collaborating with E2A, while SMARCD2 not only affects the fate trajectory of CD8^+^ T cells but also plays a key role in the development of myeloid cells. These findings provide novel perspectives for understanding the role of chromatin remodeling complexes in immune cell differentiation and function.

#### 2.2.4 SMARCA2/4

SMARCA4 and SMARCA2 are two mutually exclusive catalytic subunits of the SWI/SNF complex, both possessing a bromodomain and an ATPase domain. They are highly homologous and participate in multiple cellular processes (86). Mutations in both domains are associated with tumorigenesis and progression and are also synthetic lethal targets (87–89).

During the immature lymphocytic stage, early studies in HSCs have shown that SMARCA4 promotes the expression of KIT and MYC to support HSC development (85). Recent studies also show that SMARCA4 can bind to the promoter of KLF2A and trigger its transcriptional profile. KLF2A then promotes the production of nitric oxide (NO), and the NO microenvironment is crucial for HSC differentiation (90). Furthermore, during the development of immature T cells, multiple studies have shown that SMARCA4 promotes the formation of mature T cells by regulating MYC expression (91–93), and its loss often leads to disruption of the Wnt pathway. Additionally, acute depletion of SMARCA4 can impair the expression of key early T cell development factors such as GATA3 and T-BET, and reduce H3K27 acetylation levels in promoter and enhancer regions, as well as the binding of P300 (94). Finally, during the development of immature B cells, studies have shown that SMARCA4 collaborates with factors such as E2A and EBF1, located at super-enhancers, where these super-enhancers frequently interact with the MYC promoter region to promote the expression of MYC and accelerate precursor B cell growth (72, 95).

In mature T cells, recent studies have shown that the loss of SMARCA4 primarily improves the state of exhausted T cells. In the case of T cell activation, SMARCA4 mainly collaborates with AP-1, NFAT and NF-κB to promote the expression of IFNG and IL2. In the case of T cell exhaustion, SMARCA4 works with BATF, MYB, and others to promote the expression of TOX, TIGIT, and others. It particularly enhances chromatin accessibility in HNF1B binding regions and the loss of HNF1B leads to a reduction in the proportion of exhausted CD8 + T cells. In summary, this study highlights that treatment with SMARCA4 inhibitors enhances the persistence of CD8^+^ T cells, increases memory phenotypes, and boosts anti-tumor activity (96, 97). Most studies on Tex mechanisms have focused on CD8^+^ T cells, but recent researches have also revealed the regulatory mechanisms of SMARCA2 and SMARCA4 in the exhaustion process of effector CD4^+^ T cells. In CD4^+^ T cells, the SMARCA2-mediated SWI/SNF complex inhibits CD274 expression, and SMARCA2 also antagonizes the action of EZH2, which can promote CD274 expression. However, in the case of tumor cell-induced CD4^+^ Tex, the replacement of SMARCA2 by SMARCA4 leads to increased CD274 expression. Therefore, inhibiting EZH2 or SMARCA4 can reverse the CD4^+^ Tex state (98).

SMARCA2 and SMARCA4 also goven TH1 differentiation and function. First, SMARCA4 mediates chromatin remodeling at the IL12RB2 gene regulatory region to promote Th1 cell differentiation (99, 100). Additionally, the SMARCA2-mediated SWI/SNF complex can regulate chromatin remodeling at the promoter regions of the IFNG and TBX21 genes. WAS mutations disrupt SMARCA2 enrichment at these promoters, leading to impaired TH1 function and damaging the activation of the Notch signaling pathway and its downstream effector NF-κB (101).

In the determination of mature B cell fate, the loss or reduced function of SMARCA4 leads to decreased activity of TFs that antagonize BCL-6, such as PU.1. This causes GC cells to revert to the dark zone and become centroblasts again, reducing the number of memory B cells or plasma cells. Excessive retention of GC cells can also promote lymphoma formation (102, 103).

Lymphocytes lacking SMARCA4 rapidly undergo cell cycle arrest and apoptosis. Therefore, precise targeting of SMARCA4 in leukemia treatment may have specific therapeutic effects (104). Several studies have shown that SMARCA4 can maintain the growth of myeloid and lymphoid leukemia cells, primarily because SMARCA4 mediates the expression of MYC and PU.1 (85, 105–108). Recent research has further revealed that SMARCA4 occupies the transcriptional activation site of PPP2R1A, inhibiting its expression and activating the PI3K/AKT signaling pathway to promote the expression of oncogenes MYC and BCL2. Targeting SMARCA4 represents a promising strategy for treating adult B cell-acute lymphoblastic leukemia (109). In viral infections, SMARCA4 can also physically interact with the human T-cell leukemia virus type 1 (HTLV-1) regulatory protein HBZ to promote the downregulation of HTLV-1 transcription, thus impeding the progression of T cell leukemia (110).

In summary, during lymphocyte differentiation, SMARCA4 and SMARCA2 affect lymphocytic function and the development of related diseases, especially leukemia and lymphoma, by enhancing chromatin accessibility and interacting with various TFs. The loss of SMARCA4 and SMARCA2 can also significantly reduce the expression of exhaustion genes in T cells. These findings not only help improve immunotherapy but also provide novel targets for the therapy of hematologic diseases, particularly various types of leukemia or lymphoma.

### 2.3 PBRM1

PBRM1 is a characteristic subunit of PBAF, containing multiple bromodomains, which participate in specific chromatin recognition. The loss of PBRM1 often increases histone modification in promoter region (111, 112). Recent studies have also revealed that PBRM1 loss leads to abnormal activation of the NF-κB pathway and increased polarization and infiltration of M1 macrophages (113, 114). During HSC development, PBAF directly binds to the CDKN1A promoter region via PBRM1, negatively regulating CDKN1A expression by inhibiting its transcriptional level, thus maintaining HSC homeostasis and regulating lymphocytic differentiation (115). Meanwhile, PBRM1 can directly bind to regulatory elements in the IL10 locus to suppress IL10 expression. PBRM1-deficient Th2 cells overexpress the immunoregulatory cytokine IL-10 (116).

Hence, the loss of PBRM1 may damage the composition and function of immune cells in the tumor microenvironment, and disrupt hematopoietic homeostasis. However, further research is needed to explore the specific functions and mechanisms of PBRM1 in lymphocytes.

### 2.4 BRD7/9

Both BRD7 and BRD9 contain a bromodomain, which can recognize and bind acetylated lysine residues, particularly on histones. This domain allows BRD7 and BRD9 to coordinate PBAF and ncBAF in targeting specific histone regions to maintain chromatin openness. Bromodomain inhibitors targeting this structure are gradually developed clinically for the regimens of cancer and inflammatory diseases (117–120).

BRD7 is a characteristic subunit of PBAF, and its multiple roles in maintaining cell cycle homeostasis and its involvement in tumor suppression have gained widespread attention (121–123). T-BET is a key TF for the differentiation of short-lived effector T cells (SLECs). Recent studies have shown that BRD7 expression is enhanced in effector CD8^+^ T cells after acute viral infection. BRD7 acts as a “bridge” to cooperate with SMARCC1 and SMARCA4, helping PBAF bind to the TBX21 promoter region and upregulating TBX21 expression (124). This finding provides a new therapeutic target for diseases associated with CD8^+^ T cell dysfunction.

BRD9 is a unique recognition subunit of ncBAF. In immature B lymphocytes, the loss of BRD9 leads to a tendency of increased myeloid differentiation and enhances the binding of CCCTC-binding factor (CTCF) to chromatin, thereby enhancing chromatin loops, resulting in impaired early B cell development (125). In mature T lymphocytes, BRD9 plays an important role in Treg differentiation and function. FOXP3 is a key TF for Treg cells, and its expression level directly decides Treg suppression function (126–128). BRD9 co-localizes with key enhancers of the FOXP3 gene. BRD9 enhances FOXP3 binding to its own promoter, stabilizes FOXP3 transcription, and activates the expression of CTLA4, ICOS, and TIGIT, thus maintaining Treg function (19).

In summary, BRD7 and BRD9 are characteristic recognition subunits of PBAF and ncBAF and play important roles in targeting PBAF and ncBAF. On one hand, their bromodomains help the complex directly bind to the TBX21 promoter region; on the other hand, they collaborate with key factors such as CTCF and FOXP3 to target specific chromatin regions, providing an important molecular foundation for the differentiation and function maintenance of immature B cell, CD8^+^ T cell and Treg. These studies reveal the potential therapeutic value of BRD7/9 as key factors in lymphocyte immune regulation.

### 2.5 ACTL6A

ACTL6A is a common subunit of the SWI/SNF complex, and recent studies have identified that ACTL6A can inhibit ferroptosis in gastric cancer (129). However, research on ACTL6A in lymphocytes is very limited. Currently, it has only been described to play a role in maintaining HSC proliferation and survival. The loss of ACTL6A weakens the expression of MYC and RUNX1, interfering with the normal differentiation function of HSCs (130). The loss of ACTL6A leads to an imbalance in HSC differentiation potential, disrupting the stability of the hematopoietic system and subsequently impacting lymphocyte differentiation. Future research may further clarify the mechanisms by which ACTL6A and ferroptosis function in various lymphocyte subpopulations.

### 2.6 PHF10

PHF10 is a protein containing a PHD domain and a core component of the SWI/SNF complex. It plays an important role in gene expression regulation, cell differentiation, and tumorigenesis (131–133). Current studies have displayed that PHF10 maintains the pluripotency of HSCs via the regulation of GATA2 and myeloid differentiation genes (134). Its loss leads to impaired lymphocytic differentiation. However, the mechanisms by which PHF10 functions in various lymphocytic subpopulations need to be further clarified.

## 3 Clinical applications and treatments

**Figure 1:**
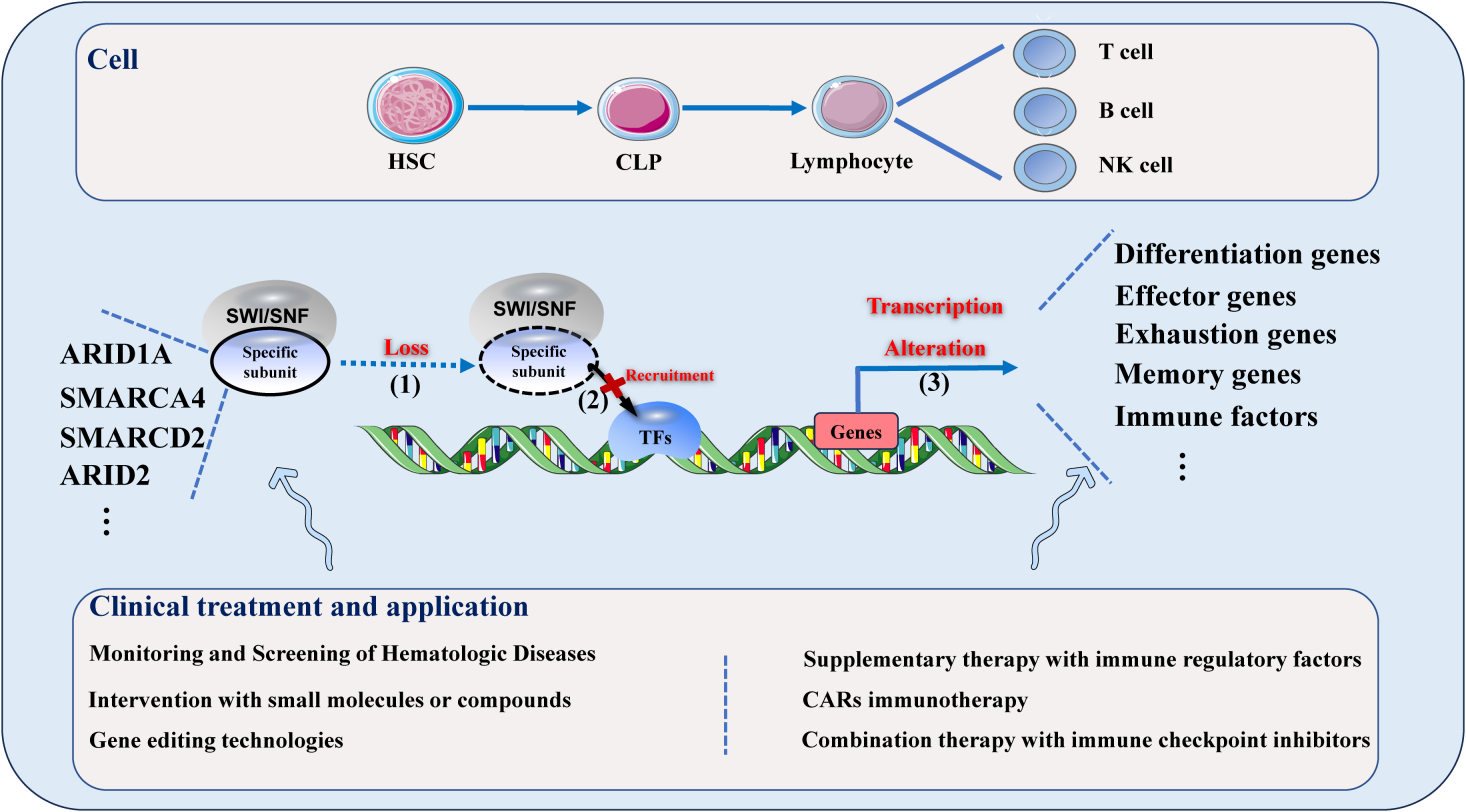
The impact of specific SWI/SNF complex subunit loss on the overall function and targeting of the SWI/SNF complex at various stages of lymphocyte differentiation, as well as clinical treatments and applications targeting these changes. The progression of the effect on lymphocytes is as follows: (1) Loss of a specific subunit of the SWI/SNF complex; (2) Loss of subunit leads to impaired targeting recruitment of the SWI/SNF complex; (3) Changes in the transcription levels of lymphocyte differentiation, effector, exhaustion, memory genes, and others.

In the context of tumors (such as leukemia and lymphoma) and immune diseases (including autoimmune diseases and infections), the expression and mutations of SWI/SNF complex subunits in lymphocytes can be monitored in the bone marrow, thymus, and peripheral blood. When mutation or loss of SWI/SNF specific subunits is detected, gene editing techniques, small molecules, or compounds can be employed to intervene in the subunit or its cooperating TFs. Additionally, appropriate immune regulatory factors can be applied in supplementary therapy targeting the downregulated genes to restore the chromatin remodeling function of the SWI/SNF complex or lymphocyte function in disease states (**Table 2**). Meanwhile, targeting the changes in lymphocyte exhaustion, effector, and memory genes can integrate these regulatory mechanisms into CARs therapy and combination therapy with immune checkpoint inhibitors. (**Figure 1**).

In solid tumors, mutations in specific subunits of the SWI/SNF complexes can accelerate the onset of various cancers and reduce immune cell infiltration in the tumor microenvironment (135). Early studies have demonstrated that mutations in genes such as ARID1A and SMARCA4 may diminish the activity of immune cells within the tumor microenvironment, thereby accelerating immune evasion by the tumor and decreasing the tumor cell responsiveness to immunotherapy (136–138). Regarding T cell regulation, dysfunction of the SWI/SNF complex may lead to reduced T cell infiltration or impaired function within the tumor, ultimately affecting the immune surveillance and clearance of tumor cells (139).

From a broader perspective, cBAF constitutes the largest proportion and exhibits the most extensive function among the three SWI/SNF complexes (20, 21). It is involved in initiating the differentiation of stage-specific genes in lymphocytes, particularly during the early differentiation stage and the terminal fate determination stage (7, 22, 30, 33–35, 40, 41, 83, 98). This supports lymphocytic differentiation, the maturation of effector functions, the formation of memory T cells, and plasma cell differentiation. Additionally, cBAF also regulates certain immune evasion mechanisms (27, 83). PBAF, aside from its role in lymphocyte differentiation, enhances the expression of effector factors in Th2 cells and the function of CD8^+^ T cells, thereby supporting immune responses, especially in chronic inflammation and tumor models (46, 55, 57, 98, 116, 117, 125, 140). In contrast, ncBAF is more inclined to promote B cell maturation and the formation of immune tolerance. Loss of ncBAF function within the immune system can lead to Treg dysfunction, which in turn can trigger the development of autoimmune diseases (19, 84, 98, 126).

**Figure 2:**
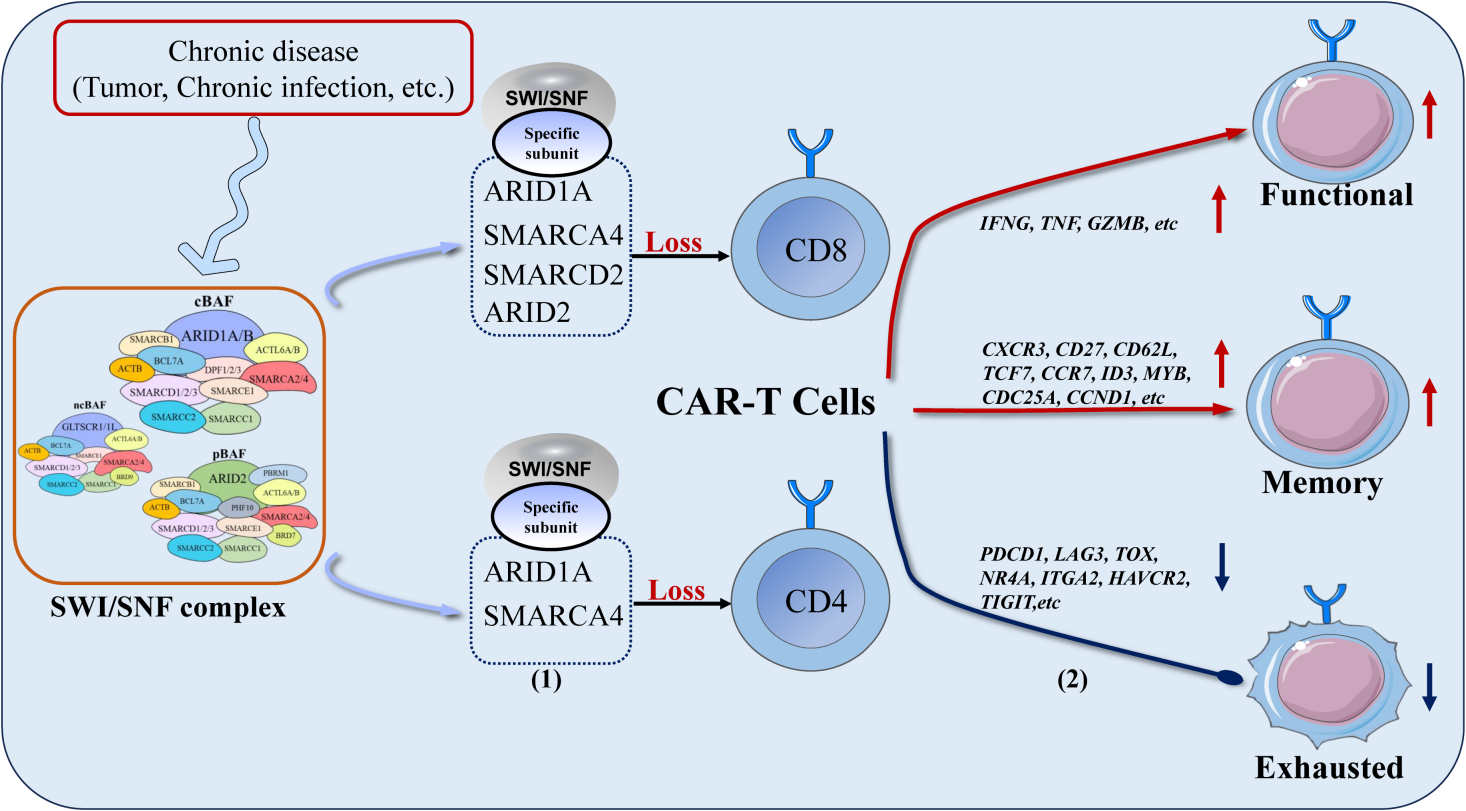
In chronic diseases, targeted intervention of specific SWI/SNF complex subunits can effectively improve the efficacy of CAR-T therapy: (1) Pre-targeted intervention of specific subunits of the SWI/SNF complex in CAR-T cells; (2) The function and persistence of CAR-T cells are enhanced after the intervention: increased expression of effector genes and memory genes in CAR-T cells, decreased expression of exhaustion genes, accompanied by increased differentiation of effector CAR-T cells and memory CAR-T cells, and decreased differentiation of exhausted CAR-T cells.

Recent studies on the SWI/SNF complex in lymphocytes have documented that it can effectively improve CARs therapy. CARs are immune therapies that employ genetic engineering to modify a patient’s own immune cells by equipping them with chimeric antigen receptors, enabling them to target and kill abnormal cells. Currently, applications such as CAR-T, CAR-Treg, CAR-NK, and CAR-M are in use (140, 141). In recent years, significant results have been achieved with CAR therapy in non-solid tumors. However, the therapeutic effect is less favorable in some solid tumors and inflammatory diseases, which is decided by the duration and effector function of immune cells. Therefore, targeting and regulating certain key genes within T cells to enhance cell persistence and efficacy is a crucial step in improving treatment outcomes (142–144). For example, targeting the knockdown of exhaustion markers such as BATF, IRF4, TOX, NR4A, TIGIT, PD-1, and TIM-3 can extend the persistence of CAR-T cells in the body (50, 145–150), while targeting the upregulation of effector genes such as C-JUN can effectively enhance the effector function of CAR-T cells (151). Targeting and regulating specific subunits of the SWI/SNF complex can both reduce multiple exhaustion genes and increase functional genes (**Table 2**). Therefore, compared to regulating individual exhaustion and functional genes, directly targeting and regulating specific subunits of the SWI/SNF complex can more effectively improve the effector and exhaustion states of CAR-T cells (**Figure 2**).

CAR-T therapy primarily involves CD4^+^ T cells and CD8^+^ T cells. The SWI/SNF complex subunits ARID1A, SMARCA2, and SMARCA4 are closely associated with the exhaustion and function of CD4^+^ T cells, especially in tumor models (33). MATR3 can cooperate with ARID1A to promote the expression of TOX, SMARCA2 can antagonize EZH2 to inhibit PD-L1 expression, while SMARCA4 interacts with EZH2 to promote PD-L1 expression (98). Therefore, in CAR-T therapy, targeting the inhibition of MATR3, EZH2, ARID1A, and SMARCA4 expression can effectively reduce CD4^+^ CAR-T cell exhaustion and enhance effector functions.

In CD8^+^ T cells, targeting the SWI/SNF complex subunits ARID1A, ARID2, SMARCA4, and SMARCD2 can effectively affect the terminal fate determination and effector functions of CD8^+^ T cells. However, different interventions are required in acute and chronic diseases. Several studies have consistently shown that loss of ARID1A, SMARCA4, and SMARCD2 reduces CD8^+^ Tex, increases memory CD8^+^ T cells, and enhances their persistence. In acute infection models, studies suggest that the loss of ARID1A, SMARCA4, and BRD7 impairs the short-term effector functions of CD8^+^ T cells, making SLEC less effective in combating acute bacterial and viral infections (7, 21, 124, 152). However, in chronic infections and tumors, SLEC cannot persist, so the focus is often on the sustainability of the immune response. The loss of ARID1A, SMARCA4, and SMARCD2 may partially decrease T cell effector function, but by promoting the expression of T cell memory phenotypes (55, 153–155), CD8^+^ T cells are less likely to enter an exhausted state under continuous antigen stimulation, thereby improving long-term antitumor or anti-infection responses (22, 97). The loss of ARID2 causes CD8^+^ T cells to convert to Tex Int, which not only partially improves effector function but also enhances T cell proliferation and survival (54, 56). Therefore, in CAR-T therapy in chronic diseases, pre-treatment of CAR-T cells to knock out ARID1A, ARID2, SMARCA4, and SMARCD2 can enhance the persistence and effector functions of CD8^+^ CAR-T cells.

CAR-Treg therapy shows promising application prospects in immune-related diseases such as autoimmune diseases and transplant rejection (156). Recent studies focus on optimizing the function and stability of CAR-Tregs, and SWI/SNF complex subunits like SMARCB1, SMARCA4, SMARCD1, ARID1A, and BRD9 are closely associated with Treg immunosuppressive function, migratory ability, and proliferative capacity (19, 59, 83). These genes may also become the focus of future CAR-Treg research, and targeting these key SWI/SNF subunits is expected to enhance CAR-Treg proliferation and function.

CAR-NK therapy is currently in the cradle of research, but it has shown unique advantages in the treatment of solid tumors (157, 158). However, research on the SWI/SNF complex in NK cells is currently limited and needs further development. Recent studies have evidenced that specific SWI/SNF complex subunits, such as BRD9, play a key role in macrophage immune responses. Targeting these key SWI/SNF subunits is also expected to enhance the immune therapeutic effect in CAR-M technology (17, 78, 159, 160).

In conclusion, targeted interventions in specific SWI/SNF complex subunits can both increase the expression of a range of lymphocyte key functional genes and decrease the expression of exhaustion genes. Applying these mechanisms in CARs technology can more effectively enhance cell persistence and function, allowing more patients to benefit from solid tumor treatments.

## 4 Perspectives and challenges

First, most research on the SWI/SNF complex has focused on *in vitro* experiments or mouse models, which differ from human treatments. Additionally, the changes in communication between immune cells after targeting SWI/SNF subunits in lymphocytes remain unclear, which is necessary to minimize treatment side effects.

Second, research on lymphocytes has mainly focused on ARID1A, SMARCA4, and SMARCA2, whereas the regulatory role of the SWI/SNF complex in NK cells has not been investigated. Future research could further explore the roles of other subunits in various lymphocytic subpopulations.

Third, future studies may further develop targets like EP400 that can compensate for the loss of SWI/SNF subunits (161).

Fourth, studies on the synthetic lethality effects of ARID1A and ARID1B, as well as SMARCA2 and SMARCA4 (162), in lymphoma and leukemia remain limited. Additionally, SMARCC1 and SMARCC2, as core scaffold proteins of the SWI/SNF complex, share structural similarities and complementary roles. Future studies could further clarify whether SMARCC1 and SMARCC2 exhibit synthetic lethality in diseases.

Fifth, in CAR-T applications, targeting different subunits of the SWI/SNF complex at various stages of T cell differentiation will have different effects. Future research needs to further clarify the timing of intervention to optimize CARs technology.

## 5 Conclusion

The SWI/SNF complex is a key regulatory factor in lymphocyte biology, crucial for lymphocytic development, differentiation, and immune function. The loss of individual subunits of the SWI/SNF complex disrupts chromatin accessibility at key regulatory regions. In immature lymphocytes, the loss of subunits results in abnormal cell differentiation and increases the risk of leukemia and lymphoma. In mature lymphocytes, the loss of specific subunits improves effector functions. Thus, targeting SWI/SNF subunits at various stages of lymphocytic development can effectively modify cell differentiation and function. Future studies that clarify the specific regulatory mechanisms of SWI/SNF subunits in different lymphocyte subpopulations are likely to accelerate the development of immunotherapy and personalized medicine. This may offer new therapeutic strategies for treating infections, cancer, autoimmune diseases and so on.
