# Supplementary material for: Chromatin remodeling in lymphocytic function and fate: the multifaceted roles of SWI/SNF complex
Source: Front Immunol. 2025 Apr 24;16:1575857. doi: 10.3389/fimmu.2025.1575857 (PMC12058788; doi:10.3389/fimmu.2025.1575857)
Supplement: Supplementary file 1 [file Table1.docx]

**Supplement Table Research methods of SWI/SNF subunits**

| Subunit | Cell | Research model | Gene intervention and gene knockout types | Gene intervention tool | Drug | Drug function | Ref |  |
| --- | --- | --- | --- | --- | --- | --- | --- | --- |
| ARID1A | HSC and CLP | Mouse model | Conditional knockout  Germline knockout | Cre-loxP system | Poly(I:C) | Inducing ARID1A knockout | (30) |  |
|  | T cell  (DN1-DN4) | Mouse model | Conditional knockout | iCRE system | / | / | (20) |  |
|  | CD8^+^ T cell | Mouse model | Conditional knockout | CRISPR-Cas9 | / | / | (22) |  |
|  | CD8^+^ T cell | Mouse model | Conditional knockout  Germline knockout | CRISPR-Cas9 | BRD-K98645985 | Inhibiting cBAF function | (21) |  |
|  | CD8^+^ T cell | Mouse model | Conditional knockout | CRISPR-Cas9  Cre-loxP system | BRM014 | Inhibiting cBAF function | (7) |  |
|  | Mature B Cell | Mouse model | Conditional knockout | Cre-loxP system | IL-1β inhibitor | Recovering the cell function after ARID1A deletion partially | (34) |  |
|  | Mature B Cell | Mouse model | Conditional knockout | CRISPR-Cas9  Cre-loxP system | FHD-286  AU-1533062 | Inhibiting the expression of SMARCA4/2 | (35) |  |
| ARID1B | HSC and CLP | Mouse model | Conditional knockout | Cre-loxP system | Poly(I:C) | Inducing ARID1B knockout | (40) |  |
|  | HSC and CLP | Mouse model | Conditional knockout  Germline knockout | Cre-loxP system | PIpC | Inducing ARID1B knockout | (41) |  |
| ARID2 | HSC and CLP | Mouse model | Conditional knockout | Cre-loxP system | Lipopolysaccharide | Activating inflammatory pathways | (45) |  |
|  | CD8^+^T cell | Mouse model | Conditional knockout  Germline knockout | CRISPR-Cas9  Cre-loxP system | Anti-PD-L1 | Inhibiting immune checkpoints | (54) |  |
|  | CD8^+^T cell | Mouse model | Conditional knockout  Germline knockout | CRISPR-Cas9  Cre-loxP system | / | / | (56) |  |
| SMARCB1 | Treg | Mouse model | Conditional knockout | CRISPR-Cas9 | MG132 | Inhibiting protein degradation | (59) |  |
|  | CD8^+^ T cell | Mouse model | Conditional knockout | Cre-loxP system | / | / | (57) |  |
| SMARCC1 | HSC and CLP | Mouse model | Transduction | Retrovirus | / | / | (71) |  |
|  | HSC and CLP | Mouse model | Conditional knockout  RNAi | Cre-loxP system siRNA | PIpC | Inducing SMARCC1 knockout | (72) |  |
|  | HSC and CLP | Mouse model | Conditional knockout | Cre-loxP system | Poly(I:C) | Inducing SMARCC1 knockout | (73) |  |
|  | TH17 | Mouse model | Conditional knockout  Transduction | Retrovirus | / | / | (77) |  |
|  | GC Tfh | Mouse model | Conditional knockout | Cre-loxP system | / | / | (81) |  |
| SMARCD1 | HSC and CLP | Mouse model | Conditional knockout | Cre-loxP system | UM171 | Improving stem cell growth | (82) |  |
|  | Treg | Mouse model | Conditional knockout  Germline knockout | CRISPR-Cas9  Cre-loxP system | PMA  Ionomycin  Pertussis toxin | Activating or inhibiting lymphocyte function | (83) |  |
| SMARCD2 | CD8^+^ T cell | Mouse model | Conditional knockout  Germline knockout | CRISPR-Cas9 | BRD-K98645985 | Inhibiting cBAF function | (21) |  |
| SMARCA4 | HSC and CLP | Human leukemia cell | RNAi | shRNA | / | / | (85) |  |
|  | HSC and CLP | Mouse model | Conditional knockout | Cre-loxP system | PIpC | Inducing SMARCA4 knockout | (91) |  |
|  | HSC and CLP | Zebrafish model | Germline knockout | TALEN technique | S-nitroso N-acetylpenicillamine | Recovering the cell function after SMARCA4 deletion partially | (90) |  |
|  | Pro-T Cell,  Pro-B Cell,  CD4^+^ T Cell | Mouse model | Conditional knockout | CRISPR-Cas9 | Trichostatin A | Inhibiting histone deacetylase | (94) |  |
|  | Pre-B Cell | Mouse model | Conditional knockout  RNAi | Cre-loxP system shRNA | 4-hydroxytamoxifen | Inducing SMARCA4 knockout | (95) |  |
|  | CD8^+^ T cell | Mouse model | Conditional knockout | CRISPR-Cas9 | CMP14  FHT-1015  ACBI1  AU-15330 | Inhibiting and degrading SMARCA4/2 | (97) |  |
|  | TH1 | Human naive CD4+ T cells | RNAi | siRNA | Cyclosporine A | Inhibiting BRG1 recruitment | (99) |  |
|  | TH1 | Human naive CD4+ T cells | Transduction  RNAi | Retrovirus  shRNA | Cyclosporine A | Inhibiting BRG1 recruitment | (10) |  |
|  | Mature B Cell | Mouse model | Conditional knockout | Cre-loxP system CRISPR-Cas9 | Doxycycline | Inducing the degradation of SMARCA4 | (102) |  |
|  | Mature B Cell | Mouse model | Conditional knockout | Cre-loxP system | / | / | (103) |  |
|  | Immature B Cell | Mouse model | Conditional knockout  RNAi | shRNA | PFI-3 | Inhibiting SMARCA4 function | (109) |  |
| SMARCA2 | TH1 | Human T-helper cells | Transfection | WASp plasmid | / | / | (101) |  |
| PBRM1 | HSC and CLP | Mouse model | Conditional knockout | Cre-loxP system | 4-hydroxytamoxifen | Inducing PBRM1 knockout | (115) |  |
|  | Th2 | Mouse model | Conditional knockout | Cre-loxP system | / | / | (116) |  |
| BRD7 | CD8^+^ T cell | Mouse model | Conditional knockout | Cre-loxP system | / | / | (124) |  |
| BRD9 | Immature B Cell | Mouse model | Conditional knockout | Cre-loxP system | BI-7273 | Inhibiting the bromine domain of BRD9 | (125) |  |
|  | Treg | Mouse model | Conditional knockout | CRISPR-Cas9 | / | / | (19) |  |
| ACTL6A | HSC and CLP | Mouse model | Conditional knockout | Cre-loxP system | / | / | (130) |  |
| PHF10 | HSC and CLP | Mouse model | Conditional knockout | Cre-loxP system | / | / | (134) |  |
